# Chronic hypoxia down‐regulates tight junction protein ZO‐2 expression in children with cyanotic congenital heart defect

**DOI:** 10.1002/ehf2.12081

**Published:** 2015-12-21

**Authors:** Emma L. Jenkins, Massimo Caputo, Gianni D. Angelini, Mohamed T. Ghorbel

**Affiliations:** ^1^ Bristol Heart Institute, School of Clinical Sciences University of Bristol Bristol UK

**Keywords:** ZO‐2, Hypoxia, Congenital heart defects, Cyanosis, Cardiomyocyte

## Abstract

**Aims:**

Tight junction protein zonula occludens protein 2 (ZO‐2) is a member of the membrane‐associated guanylate kinases protein family known to be expressed at tight junctions of epithelial and endothelial cells and at adherens junctions (AJs) in cardiomyocytes. Little is known about ZO‐2 expression and function in the human heart. Here, we examined the hypothesis that chronic hypoxia down‐regulates ZO‐2 expression in human myocardium and cultured rat cardiomyocytes.

**Methods and results:**

Patients with a diagnosis of cyanotic (*n* = 10) or acyanotic (*n* = 10) Tetralogy of Fallot undergoing surgical repair were used to examine ZO‐2 messenger RNA and protein expression by real time‐PCR, immunohistochemistry, and western blotting. A model of cultured rat cardiomyocytes was used to measure ZO‐2 and AJ proteins levels in response to hypoxia and to investigate ZO‐2 cellular localization. We showed that ZO‐2 is expressed in myocardial tissue in acyanotic and cyanotic children with congenital heart defects. ZO‐2 was specifically down‐regulated in cyanotic myocardium at both the messenger RNA and protein levels when compared with acyanotic patients. This specific down‐regulation can be mimicked in cultured rat cardiomyocytes by treating them with hypoxic conditions confirming that ZO‐2 gene down‐regulation is specifically due to cyanosis. Furthermore, in addition to its cytoplasmic expression, ZO‐2 showed nuclear expression in cultured rat cardiomyocytes suggesting potential role in transcription regulation.

**Conclusions:**

Hypoxia down‐regulates ZO‐2 expression in both cyanotic patient's myocardium and cultured rat cardiomyocytes. This down‐regulation suggest an involvement of ZO‐2 in cardiac remodelling of AJs in cyanotic children and may explain the greater susceptibility of cyanotic patients to corrective heart surgery.

## Introduction

Congenital heart defects (CHDs) account for the largest number of birth defects in humans, with an incidence of ~ 8 per 1000 live births (British Heart Foundation data 2004), and can lead to heart failure. Tetralogy of Fallot (TOF) is a common form of cyanotic CHD, with an incidence rate of 1 out of 2000 newborns. Currently, primary correction at a young age is the treatment of choice. However, corrective surgery could result in an ischaemia/reperfusion injury.

Cyanotic paediatric patients undergoing cardiac surgery are exposed to a chronic hypoxic state that can reduce their antioxidant reserve capacity, leading to a greater susceptibility to the oxidative stress of ischaemia and reperfusion at the time of surgical correction.^1^, ^2^, ^3^ Although corrective surgery of heart defects is successful, there is an increased risk of morbidity and mortality in cyanotic children compared with acyanotic.^4^ We recently examined the effect of chronic hypoxia on the transcriptomic profile in patients with TOF using gene microarray.^5^ Out of the differentially expressed genes, zonula occludens protein 2 (ZO‐2) transcript level showed a significant decrease in cyanotic patients compared with acyanotic. ZO proteins (ZO‐1, ZO‐2, and ZO‐3) belong to the membrane associated guanylate kinase (MAGUK)‐like protein family characterized by containing PDZ [post synaptic density protein (PSD95), *Drosophila* disc large tumor suppressor (Dlg1), and zonula occludens‐1 protein (zo‐1)], Src homology 3, and guanylate kinase domains. ZO‐2, also named tight junction protein 2, is expressed at tight junctions (TJs) of epithelial and endothelial cells, which are membrane structures that regulate the passage of ions and molecules through the paracellular pathway. ZO‐2 is a structural protein, which links transmembrane TJ proteins to the actin cytoskeleton and was first identified as a 160 kDa protein, which co‐immunoprecipitates with ZO‐1.^6^ ZO proteins dimerize at the TJ and form ZO‐1/ZO‐2 and ZO‐1/ZO3 complexes.^7^ ZO‐1/ZO‐2 are highly homologous proteins but differ at the C terminal where ZO‐2 contains an alternatively spliced region containing a *β*‐motif. Both proteins have been shown to bind with other TJ expressed proteins: occludin and *α*‐catenin *in vitro.* 8 ZO proteins have unique motifs not common in other MAGUK proteins, nuclear localization signals,^9^ and nuclear export signals.^10^ Additionally, recent data indicate that ZO‐2 nuclear or cell surface localization is strongly dependent on the state of cell–cell contact.^9^

Furthermore, increased nuclear staining has been observed when epithelial cells were subjected to environmental stress such as heat shock or chemical insult.^11^ Nuclear ZO‐2 has been shown to bind to a number of signalling molecules including FBJ murine osteosarcoma viral oncogene homolog (FOS), jun proto‐oncogene (JUN), and CCAAT/enhancer binding protein (C/EBP).^12^

Data from ZO‐2 down‐regulation studies show it has significant structural importance in the cell. Small interfering RNA silencing of ZO‐2 alters the gate function of TJs, the fencing properties, and actin distribution at the membrane of epithelial cells.^13^ ZO‐2 knock‐out mice die during development showing that ZO‐2 is critical for mammalian development.^14^

Very little is known about human ZO expression. Northern blotting experiments performed on adult human epithelial tissue showed ZO‐2 gene isoforms A and C are tissue specific, and both are expressed in the adult human heart.^15^ A later study showed ZO‐1 is implicated in end stage human failing hearts.^16^ ZO‐1 down‐regulation was observed at intercalated discs, which coincided with reduced levels of Cx43, a gap junction protein.^16^ The present study is the first to show ZO‐2 expression and deregulation in cyanotic and acyanotic children's myocardium at both the gene and protein level. We specifically show a down‐regulation of ZO‐2 messenger RNA (mRNA) and protein expression in patients undergoing corrective surgery for cyanotic heart defects compared with acyanotic patients. The expression of other AJ proteins was not affected by ZO‐2 down‐regulation. This can be mimicked experimentally by treating postnatal rat cardiomyocytes with hypoxic conditions. These data suggest that ZO‐2 down‐regulation contributes to cardiac remodelling of AJs in cyanotic children but not in acyanotic patients, which coincides with cyanotic children being more susceptible to reoxygenation injury post corrective surgery when compared with acyanotic children.

## Methods

### Antibodies

All antibodies used in this study are as follows: anti‐ZO‐1 (Zymed 33‐9100), anti‐ZO‐2 (Lifespan Biosciences LS‐C26439), anti‐N‐cadherin (BD Transduction Laboratories 610920), anti‐*β*‐catenin (Sigma C2206), anti *α*‐catenin (Zymed 13‐9700), anti‐*β*‐actin (Sigma A5441), and anti‐glyceraldehyde 3‐phosphate dehydrogenase (GAPDH) (Cell Signalling 14C10).

### Children myocardium biopsies

The collection of human right ventricle specimens used in this study was approved by the North Somerset and South Bristol Research Ethics Committee (REC reference 07/H0106/172), The National Research Ethics Service, England. Parental informed written consent was gained for all patients. Patients with a diagnosis of cyanotic or acyanotic TOF undergoing surgical repair at the Bristol Royal Hospital for Children were studied. Twenty patients with a diagnosis of cyanotic (*n* = 10) or acyanotic (*n* = 10) TOF undergoing surgical repair at the Bristol Royal Hospital for Children were studied. All patients were in stable condition without preoperative respiratory or inotropic support. Patients with cyanotic TOF presented with several episodes of spells and repeated saturation measurements of less than 90% (mean, 78.9 ± 6.6%), whereas patients with acyanotic TOF did not have a history of cyanotic spells and presented with saturations of greater than 90% (mean, 94.7 ± 2.2%).

Myocardium biopsies were collected from 10 acyanotic and 10 cyanotic children immediately after institution of cardiopulmonary bypass (CPB). This collection time‐point during the CPB excludes the different CPB‐related acute ischemic myocardial injury effect in these two groups. Biopsies for protein extraction were collected in Eppendorfs and snap frozen in liquid nitrogen and stored long term at −80°C. Once thawed tissue was homogenized and lysed in lysis buffer [Tris 10 mM pH 7.5, EDTA 10 mM, NaCl 150 mM, triton X100 1%, SDS 0.1% and 1% proteases inhibitor cocktail (Roche 04693116001)] and centrifuged at 1400 rpm for 15 min (*n* = 5 for both acyanotic and cyanotic). Samples for RNA isolation were collected in ribonuclease free Eppendorfs containing RNAlater solution™ (Qiagen 76104) and stored at 4°C until RNA extraction performed (*n* = 4 for both acyanotic and cyanotic).

### Rat cardiomyocyte cell culture and treatment

All the procedures involving laboratory animals conformed to the guidelines and regulations of the University of Bristol and the United Kingdom Home Office. Cardiomyocytes were prepared from neonatal (P1–2) Wistar rat myocardium cells as described previously.^17^ Cells were grown *in vitro* for 3 days, then treated with hypoxia for 24 h by incubating in a hypoxic chamber containing 5% CO_2_ and 0.2% O_2_ at 37°C. Untreated cells used as a control were cultured in a humidified incubator with 5% CO_2_ at 37°C.

### RT and real time PCR

Ribonucleic acid was isolated from human myocardium samples using RNeasy Micro Kit (Qiagen 74004) and reverse transcribed using Retroscript® kit (Ambion AM1710). Two microlitre total complementary DNA (cDNA) was used in the real‐time‐PCR reaction, which contained the following: 2x SYBR® green (ABgene), Quantitect Primer assay specific for ZO‐2 (133 bp) or 18 s (103 bp) (Qiagen QT00010290 and QT00199374, respectively). Total solutions were loaded into capillaries and run for 50 cycles at an annealing temperature of 50°C in a Lightcycler 3 (Roche). Each sample was run in duplicate, and ZO‐2 levels were normalized to 18 s. PCR end products were run on a 1% agarose gel to confirm an amplified DNA fragment of the correct size.

### Western blotting

Protein samples were prepared by adding 4x Laemmli buffer (0.24 M Tris pH 6.8, 6% SDS, 40% sucrose, 0.04% bromophenol blue and 10% *β*‐mercaptoethanol), heating to 95°C for 5 min and were loaded on a 8–10% SDS gel. Separated proteins were transferred to Hybond™ nitrocellulose membrane (Amersham RPN303D), which were subsequently blocked in 5% non‐fat dry milk/TBS‐T (TBS‐T; 20 mM Tris pH 7.4, 1.37 M NaCl, 1% Tween) for 1 h and incubated in primary antibodies overnight at 4°C. Membranes were washed three times in TBS‐T then incubated in appropriate anti‐rabbit or anti‐mouse secondary antibody (Amersham NA934 and NA931, respectively) for 1 h at RT. Membranes were washed three times in TBS‐T, and antibody bound horseradish peroxidase (HRP) was detected using ECL (Amersham RPN2132 or RPN2134); membranes were exposed to Hyperfilm^TM^ (Amersham # 28906837). Protein bands were quantified using NIH Image J software.

### Immunohistochemistry

Right ventricular specimens were fixed in 4% paraformaldehyde, washed in phosphate‐buffered saline (PBS), embedded in paraffin, and 4 µm sections were obtained. Immunohistochemistry was performed using the ABC‐Kit from Dakocytomation. Slides were observed with an Olympus B40 microscope. Pictures were taken using a Media Cybernetics camera (Bethesda, MD, USA) and pro‐image plus software (Bethesda).

### Immunocytochemistry

Rat cardiomyocytes grown on coverslips were fixed for 15 min in 3% PFA and washed three times in PBS. Coverslips were incubated in NH_4_Cl for 10 min and blocked in 10% HS for 20 min. Coverslips were then incubated in primary antibodies overnight at 4°C and the following day washed three times in PBS and incubated in secondary antibodies: CY2 and CY3 (Jackson Labs) for 1 h at RT. After washing three times in PBS, slides were mounted with Vectorshield Mounting medium containing DAPI (# H‐1200 Vector Laboratories). Fluorescent cell imaging was achieved using a Zeiss LSM 510 Meta confocal microscope (Carl Zeiss Ltd, Cambridge, UK).

### Statistical analysis

All data were analysed using the software Instat 3.1 (GraphPad). Results are expressed as ± Standard Error of Mean (±SEM). The normality of the population was assumed. Statistical significance was assessed by Student's *t*‐test. A value of *P* < 0.05 was considered to be statistically significant.

## Results

Clinical data are summarized in *Table* 1. The two groups were comparable in terms of age. None of the patients recruited in this study had a previous palliative surgery. There were no deaths and no major morbidity in both groups. CPB time was longer in the cyanotic group, who required more extensive trans‐annular patching and reconstruction of the pulmonary artery compared with the acyanotic patients.

**Table 1:** Baseline patients' characteristics and clinical outcomes

	Cyanotic (*n* = 10)	Acyanotic (*n* = 10)	*P*‐value
Age (months)	5.7 ± 2.1	6.0 ± 1.8	0.73
Pre‐operative O_2_ saturations (%)	78.9 ± 6.6	94.7 ± 2.2	0.001
Cross‐clamp time (min)	75.1 ± 11.3	54.4 ± 11.8	0.001
CPB time (min)	127.3 ± 18.4	88.1 ± 13.4	0.001
In‐hospital mortality	0	0	—
Previous surgery	0	0	—
Use of preoperative *β*‐blockers	5	1	—

Data are mean ± standard deviation.

#### ZO‐2 protein is expressed in human heart sections of both cyanotic and acyanotic congenital heart patients

**Figure 1:**
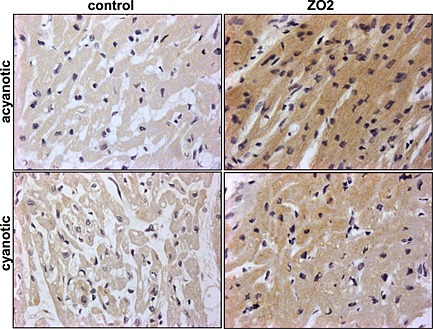
Expression of zonula occludens protein 2 (ZO‐2) in acyanotic and cyanotic paediatric myocardium tissue. Immunohistochemical analysis of paraffin embedded heart tissue from an acyanotic and cyanotic patient using ZO‐2 specific antibody. 3,3’‐Diaminobenzidine (DAB) staining reveals expression of ZO‐2 in both tissues when compared with controls, with a visible decrease in expression in cyanotic tissue compared with acyanotic. Magnification: x200.

Immunohistochemical analysis of fixed paediatric heart biopsy sections showed expression of ZO‐2 in heart tissue taken from both acyanotic and cyanotic patients undergoing corrective surgery for CHD (*Figure* 1).

#### ZO‐2 mRNA expression is down‐regulated in cyanotic myocardium compared with acyanotic patients

**Figure 2:**
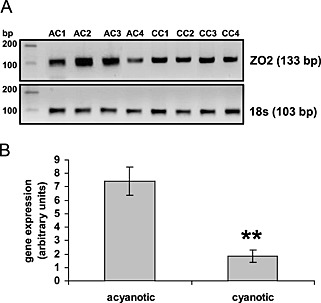
**Down‐regulation of zonula occludens protein 2 (ZO‐2) messenger RNA (mRNA) expression in cyanotic compared with acyanotic patients.** Total RNA isolated from myocardium biopsies, reverse transcribed, and PCR performed on complementary DNA using primers for ZO‐2 and 18 s. A. PCR products run on agarose gel [lanes 1–4 acyanotic samples (AC), lanes 5–8 cyanotic samples (CC)]. B. Ratio of AC mRNA copy number (arbitrary units) compared with CC showing a down‐regulation of ZO‐2 in CC children compared with AC, all values normalized to 18 s mRNA levels. Data are mean ± SEM ** = *P* < 0.01 (*n* = 4).

Total RNAs were extracted from the biopsies and reverse transcribed to produce cDNA. Realtime‐PCR analysis of human cDNA samples using ZO‐2 specific primers showed a significant down‐regulation of ZO‐2 mRNA expression in cyanotic (*n* = 4) compared with acyanotic patients (*Figure* 2).

#### ZO‐2 protein expression is down‐regulated in cyanotic patients compared with acyanotic

**Figure 3:**
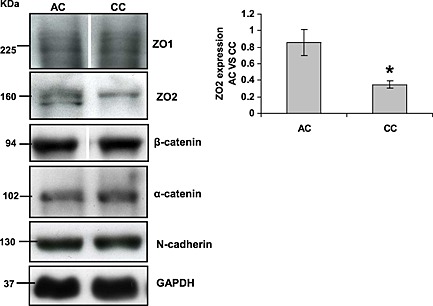
**Down‐regulation of zonula occludens protein 2 (ZO‐2) protein expression in myocardium of cyanotic compared with acyanotic patients.** Myocardium biopsies were lysed to isolate protein content and western blot analysis performed probing for ZO‐2, ZO‐1, N‐cadherin, *β*‐catenin, and GAPDH. ZO‐2 protein expression was significantly down‐regulated in cyanotic biopsies compared with acyanotic, whereas no difference in any other adherens junction expressed proteins was detected. All results were normalized to glyceraldehyde 3‐phosphate dehydrogenase (GAPDH) levels. Data are mean ± SEM * = *P* < 0.05 (*n* = 5).

Western blot analysis of protein extracted from children's myocardium biopsies reveals a specific down‐regulation of ZO‐2 protein expression in cyanotic patients (*n* = 5), coinciding with the down‐regulation of ZO‐2 at the mRNA level. To see if this was a specific down‐regulation of the ZO‐2 protein or a result of altered expression of all AJ expressed proteins, we also probed for a number of AJ expressed proteins: ZO‐1, N‐cadherin *α*‐catenin and *β*‐catenin. The levels of all other AJ expressed proteins were not significantly affected (*Figure* 3).

#### Hypoxic conditions produce a down‐regulation of ZO‐2 in cultured rat cardiomyocytes

**Figure 4:**
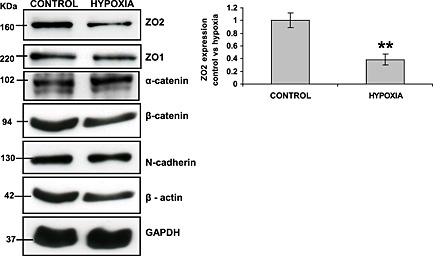
**Down‐regulation of zonula occludens protein 2 (ZO‐2) levels in rat cardiomyocytes following 24 h hypoxic treatment.** Cells were treated with hypoxic conditions by incubating in a chamber with 5% CO_2_/ 0.2% O_2_. After 24 h, cells were lysed, and western blot analysis performed probing for ZO‐2, ZO‐1, N‐cadherin, *β*‐catenin, and *β*‐actin, normalizing all results to glyceraldehyde 3‐phosphate dehydrogenase (GAPDH) levels. ZO‐2 protein expression was significantly down‐regulated in hypoxic treated cells compared with untreated cells (control), whereas no difference in ZO‐1, N‐cadherin, *β*‐catenin, or *β*‐actin levels was detected between treated and untreated cells. Data are mean ± SEM ** = *P* < 0.01 (*n* = 4).

An *in vitro* model of cyanosis would be advantageous as human biopsies are not widely available. To mimic the low‐oxygen conditions seen in cyanotic children, isolated rat cardiomyocytes grown in culture were subjected to 24 h hypoxia in a hypoxic chamber. After 24 h, cells were lysed and western blotting performed. A significant down‐regulation of ZO‐2 protein expression was observed compared with control cells, which were cultured in a humidified incubator with 5% CO_2_ at 37°C (*Figure* 4). As with children's samples, no other components of AJs junctions (ZO‐1, actin, *β*‐catenin, α‐catenin, and N‐cadherin) were significantly affected following hypoxia, showing that ZO‐2 down‐regulation is independent of other AJ proteins.

#### ZO‐2 shows a cytoplasmic and nuclear localization in neonatal heart cardiomyocytes

**Figure 5:**
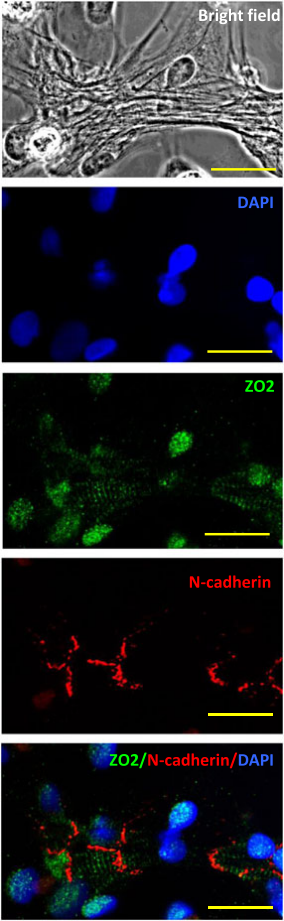
**Localization of zonula occludens protein 2 (ZO‐2) in cultured rat cardiomyocytes.** Cells were cultured for 5 days *in vitro* then fixed and stained with specific antibodies. Cardiomyocytes were stained for ZO‐2 (green) and N‐cadherin (red) then counterstained for nuclei with 4’,6‐diamidino‐2‐phenylindole (DAPI) (blue). ZO‐2 shows cytoplasmic and nuclear expression. N‐cadherin shows adherens junction expression. Bar = 20 µm.

Immunocytochemistry analysis of neonatal rat cardiomyocytes showed mainly cytoplasmic and nuclear expression of ZO‐2 (*Figure* 5). In addition, some ZO‐2 expression is also observed close to AJ (*Figure* 5).

## Discussion

In this study, we report ZO‐2 protein expression in human paediatric heart myocardium tissue. ZO‐2 has been shown to be expressed at AJs in the adult rat heart.^8^ However, to our knowledge, no other previous study has shown expression of ZO‐2 protein in the human paediatric congenital heart. ZO‐2 is expressed in the myocardium of both cyanotic and acyanotic children. Here, we show that ZO‐2 mRNA expression level is down‐regulated in cyanotic myocardium compared with acyanotic patients. This result confirmed our previous findings by microarray analysis.^5^

Our western blotting data reveal a specific down‐regulation of ZO‐2 protein expression in cyanotic patients, correlating with the down‐regulation of ZO‐2 mRNA level. All the other AJ proteins examined showed no alteration by chronic hypoxia.

In an *in vitro* cell model mimicking cyanosis, chronic hypoxia produced a down‐regulation of ZO‐2 protein in a similar way to that obtained in the human biopsies. This model would allow future functional studies into the mechanisms underlying ZO‐2 down‐regulation by hypoxia to be performed and to investigate means to restore ZO‐2 expression to normal levels. Incubating cardiomycytes for 24 h in a hypoxic chamber used in this study is a widely used protocol to induce hypoxia in rat cardiomyocytes.^17^

A down‐regulation of ZO‐1 following hypoxia has been shown to affect cell membrane junction structures of other organs.^18^ In the murine brain, TJ organization is disrupted, following hypoxia, causing an increase in permeability in the blood brain barrier.^18^ Additionally, it has been shown, in mouse, that ischemia/reperfusion injury decrease the expression of ZO‐2 in the cytoskeleton, and ischemic preconditioning preserves the integrity of tight junction structure and function in coronary endothelial cells.^19^ Our study suggests that ZO‐2 is a potential protein implicated in cardiac remodelling in children with cyanotic heart defects that could contribute to long‐term myocardial dysfunction and heart failure in cyanotic patients compared with acyanotic. AJ disruption in myocardium of cyanotic children may contribute to reoxygenation injury in cyanotic patients following CPB used during surgery. AJ disruption is seen in cardiomyocytes and endothelial cells following CPB in pigs.^20^

The nuclear localization of ZO‐2 and interaction with transcription factors^12^ provide evidence for a dual role for the protein; as a structural organizer at the plasma membrane linking TJs to the cytoskeleton and an involvement in transcriptional regulation and regulating gene expression.

In conclusion, hypoxia down‐regulates ZO‐2 expression in both cyanotic patients' myocardium and cultured rat cardiomyocytes. This down‐regulation evokes an involvement of ZO‐2 in cardiac remodelling of AJs in cyanotic children and may explain the greater susceptibility of cyanotic patients to myocardial reoxygenation injury seen during corrective heart surgery.

## Acknowledgements

We would like to thank Drs Andrew Parry and Amir Mokhtari for assisting with biopsies' collection. We are grateful to Professors David Murphy and Sarah George for reading this manuscript and valuable comments.

## Conflict of Interest

The authors declare that they have no competing interests.

## Funding

This research was funded by the British Heart Foundation, Garfield Weston Trust, and NIHR Bristol Biomedical Research Unit in Cardiovascular Medicine. M.T.G. was supported by an Intermediate Research Fellowship from the British Heart Foundation.
